# A case of advanced rectal cancer with rectovesical and ileal fistulae that developed hyperammonemic encephalopathy

**DOI:** 10.1186/s40792-015-0088-3

**Published:** 2015-09-24

**Authors:** Masahiro Maruyama, Yoshiaki Miyasaka, Atsushi Takano, Masayuki Inoue, Kazushige Furuya, Hidemitsu Sugai, Masao Hada, Hiroshi Nakagomi

**Affiliations:** Department of Surgery, Yamanashi Prefectural Central Hospital, Fujimi1-1-1, Kofu, Yamanashi, Japan

**Keywords:** Hyperammonemic encephalopathy, Rectal cancer, Rectovesical and ileal fistulae

## Abstract

Hyperammonemic encephalopathy is rarely caused by a urinary diversion. We herein experienced a case of rectal carcinoma with rectovesical and ileal fistulae that developed hyperammonemic encephalopathy. A 72-year-old man suffered from a fever, diarrhea, pneumaturia, and fecaluria beginning in April 2013 and was referred to our hospital in May 2013. He developed a loss of consciousness and whole body cramping on the first hospital day. The laboratory data indicated an inflammatory reaction and hyperammonemia with a highly elevated serum ammonia (NH3) level of 703 μg/dl.

The patient was diagnosed to have rectal carcinoma with rectovesical and ileal fistulae according to computed tomography (CT) and a water-soluble contrast enema.

We administered a solution of branched chain amino acids (BCAA) and antibiotics. Furthermore, we repeatedly irrigated bladder through the urethral catheter. The patient’s symptoms recovered, and the serum ammonia levels on the second and third hospital day were decreased to 210 and 135 μg/dl, respectively. However, the symptoms of infection and confusion were suspected to repeat; we elected to perform surgical treatment. An ileal disconnection with ileocecal bypass and sigmoidostomy were effective for preventing hyperammonemic encephalopathy.

## Background

Hyperammonemic encephalopathy had been recognized in hepatic disease, since the initial studies of portacaval shunting by Eck in 1877 [1], while non-hepatic hyperammonemic encephalopathy have been reported as rare cases, urea cycle disorder [2] medication effect such as cancer chemotherapeutics [3, 4] or valproic acid [5], and urinary tract infection [6] and after urinary tract diversion [7].

Hyperammonemia due to rectovesical fistula caused by rectal cancer is extremely rare. We could find only one case report in the Japanese literature [8]. Therefore, the pathologic physiology may be presumed according to the reports of ureterosigomoidostomy due to bladder or prostate cancer [9].

We herein report a rare case of hyperammonemic encephalopathy caused by advanced rectal cancer with rectovesical and ileal fistulae.

## Case presentation

**Fig. 1:**
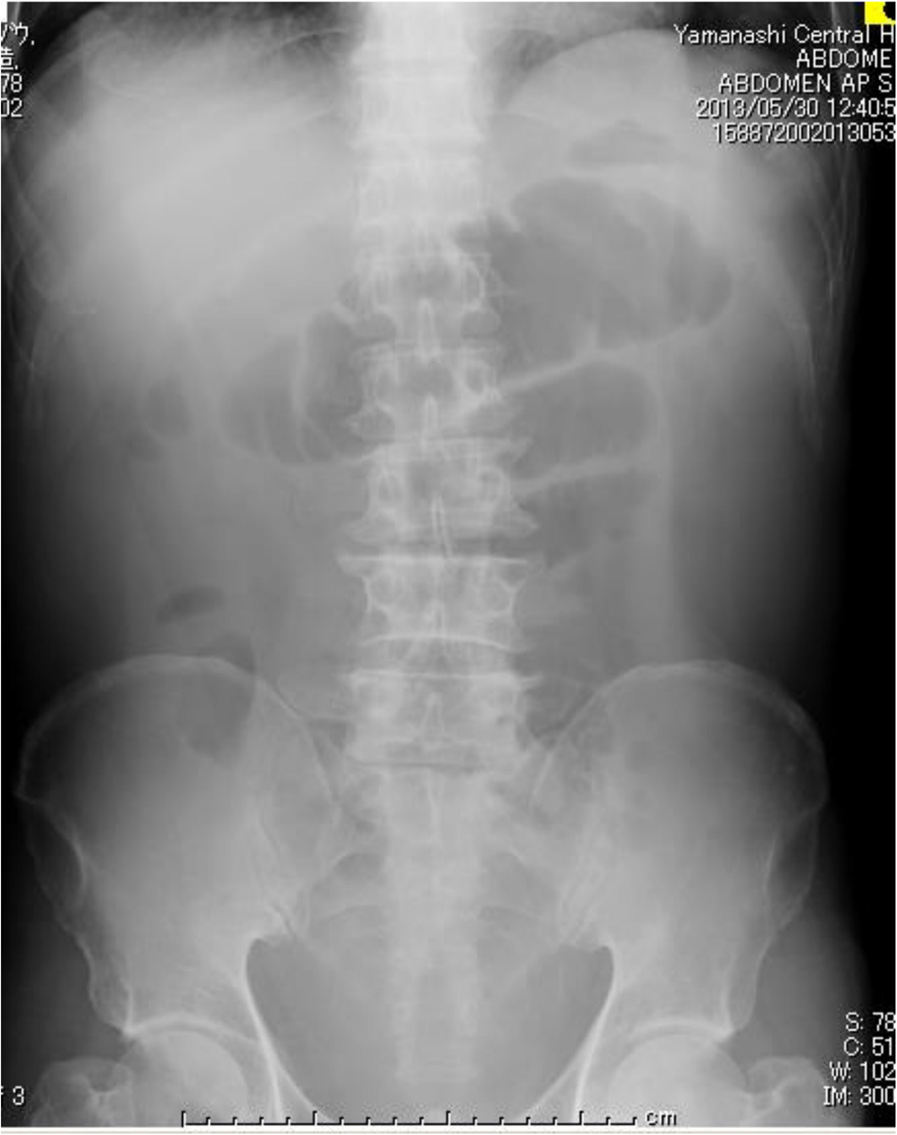
Abdominal X-ray on admission. The X-ray showed dilated small intestine indicating mechanical bowel obstruction

A 72-year-old man suffered from a fever, diarrhea, pneumaturia, and fecaluria beginning in April 2013 and referred to our hospital in May 2013. He was hospitalized due to the symptom of abdominal distension. The abdominal X-ray showed dilated small intestine indicating mechanical bowel obstruction (Fig. 1). The night of first hospital day, he developed a loss of consciousness, and the next morning, he developed whole body cramping. The patient had no previous history of hepatic disease and no episodes of confusion or neurological disorders. He was no taking any medications for any diseases.

The laboratory data indicated an inflammatory reaction with the elevation of the white blood cells count of 23,600/μl and a C-reactive protein concentration of 7.33 mg/dl and hyperchloremic acidosis, with Na 144 mEq/l, K 3.1 mEq/l, Cl 116 mEq/l, pH 7.116, PO2 145 mmHg, PCO2 34 mmHg, BE −16.8 μmol/l, and HCO3^−^ 11.3 μmol/l. Furthermore the serum ammonia (NH3) level was highly elevated to 703 μg/dl.

The level of tumor markers, CEA and CA19-9, were elevated at 7.9 ng/ml and 43.6U/ml, respectively. The liver and renal functions were normal. The culture of urine indicated the existence of enterococcus faecalis and pseudomonas aeruginosa.

**Fig. 2:**
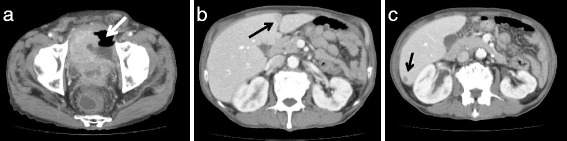
The findings of contrast computed tomography (CT). The huge tumor was occupying in minor pelvic space and invading to the bladder and small intestine. *White arrow* indicates the urinary bladder with tumor invasion and air-fluid level. **a** *Black arrows* indicated liver metastases with the size of 1.1 cm (**b**) and 1.2 cm (**c**)

Contrast computed tomography (CT) indicated the huge tumor occupying minor pelvic space which was suspected to invade the ureter and urinary bladder (Fig. 2a). Two liver metastases with the sizes of 1.1 and 1.2 cm were also observed (Fig. 2b and c).

**Fig. 3:**
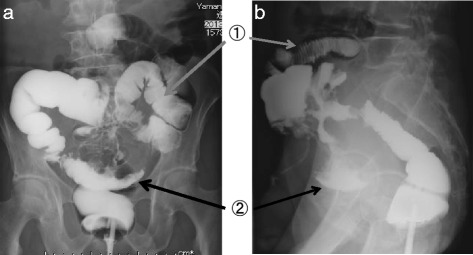
The findings of water-soluble contrast enema. Whole surrounding stenosis at rectosigmoidal junction indicated rectal cancer which was forming fistulae with ileum ① and urinary bladder ② (**a**, **b**)

**Fig. 4:**
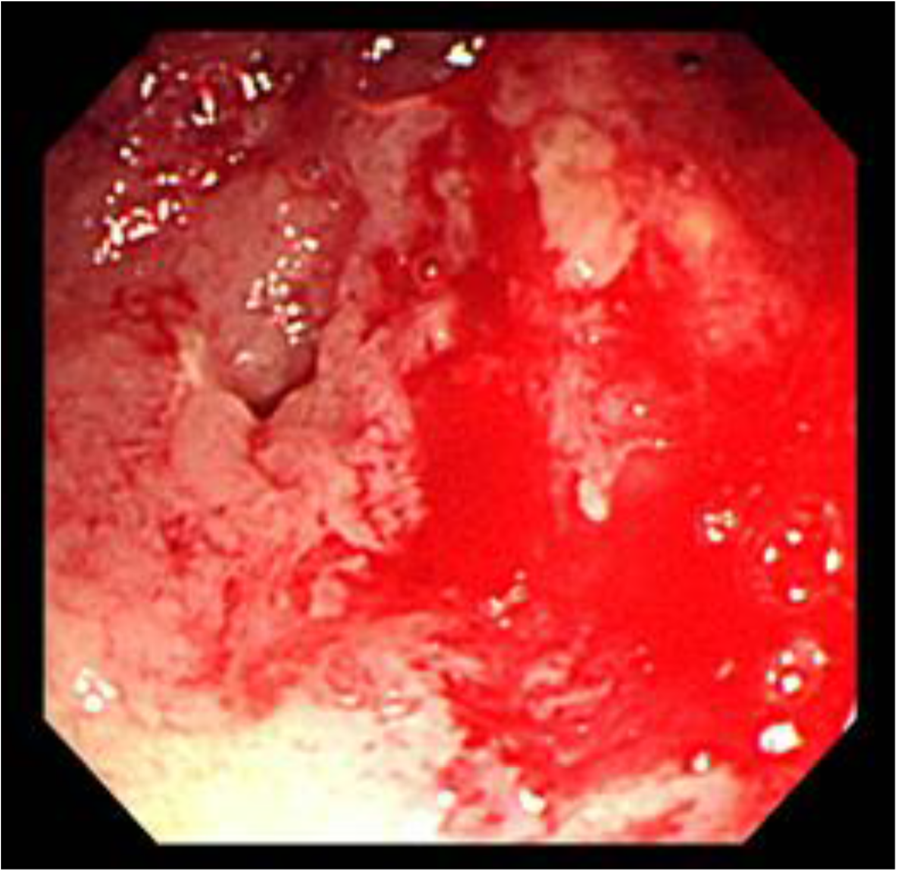
Colonoscopic finding. Whole surrounding cancer was seen at 15 cm far from anal verge. Biopsy indicated well-differentiated adenocarcinoma

A water-soluble contrast enema showed rectovesical fistulae as well as ileal fistulae (Fig. 3a, b). Colonoscopy indicated the presence of a rectal carcinoma occupying total space at 15 cm far from the anal verge (Fig. 4). The biopsy of the tumor indicated well differentiated adenocarcinoma.

We administered a solution of branched chain amino acids (BCAA) and antibiotics. Furthermore, we repeatedly irrigated the bladder through the urethral catheter. The patient’s symptoms recovered, and serum ammonia levels on second and third hospital day were decreased to 210 and 135 μg/dl, respectively. However, the symptoms of infection and confusion were suspected to repeat; we elected to perform a surgical treatment.

On the operative findings, ascites and disseminated tumors were not observed. However, the tumor could not be resected due to the invasive growth in the minor pelvic space. The surgical procedure we adopted was as follows. We disconnected both oral and anal sides of ileum which was involved in the tumor and made an ileocecal bypass. Furthermore, we performed sigmoidostomy to prevent feces pouring into the urinary bladder.

We continued irrigation of urinary bladder through the bladder catheter after the operation. The postoperative clinical course was no eventful. Serum ammonia values had been kept under 120 μg/dl.

He was treated with *XELOX* (oxaliplatin/capecitabine) for 11 cycles. However, massive ascites had been developed 1 year after the operation, and the patient died from peritoneal metastases.

The vesicular catheter had been kept during the whole period after operation, and hyperammonemia had never occurred.

### Discussions

It has been noted that a urinary diversion may sometimes be the cause of non-hepatic hyperammonemic encephalopathy [9]. However, the frequency of this complication is not known and considered to be extremely rare. Some cases have been reported in patients who underwent a urinary diversion due to bladder or prostate cancer [10]. To the best of our knowledge, only one case with rectal cancer was previously reported in the Japanese literature, in which the patient suffered from a rectovesical fistula and liver dysfunction due to multiple liver metastases [8].

Approximately 10 % (17/178) of the patients who underwent surgical treatment to create an ileal conduit developed hyperchloremic acidosis according to the report by Schmitt et al. [11]. However, hyperammonemic encephalopathy was not observed in several reviews on the complications of urinary diversion [12–14]. Urea-splitting urinary tract infection has been noted to be crucial for the development of hyperammonemic encephalopathy [15].

An elevation of the blood ammonia levels may result from the diversion of the urinary stream to the intact intestine, which provides an additional amount of ammonia that must be absorbed, especially when there is urine stasis in the bowel. Urea in the bowel is converted by urea-splitting bacteria to form ammonia. Ammonia chloride is generally reabsorbed and broken down to liberate free ammonia and hydrochloric acid. In the absence of liver dysfunction, the additional ammonia can be managed by the liver; however, in a damaged liver, the extra ammonia cannot be managed, resulting in hyperammonemia. Even with a normal liver function, urease-producing bacteria may form large amounts of ammonia, thereby, leading to hyperammonemia.

The sudden onset of hyperammonemic encephalopathy in the present case was caused by two mechanisms: urinary stasis and infection, which were induced by bowel obstruction.

Regarding the treatment of hyperammonemic encephalopathy, a bladder catheter was required to prevent urinal stasis and irrigation of bladder and antibiotics and hyperammonemia-reducing drugs were administered. However, surgical treatment is ultimately required to prevent the occurrence of repeated infection and hyperammonemia.

Concerning the surgical procedures, we could not divide the cystic bladder from rectum because of the invasive growth of the tumor. We performed surgical management only for bowel obstruction, ileal disconnection with ileocecal bypass, and sigmoidostomy. Therefore, the serum ammonia value was kept slightly high around 80~120 μg/dl after surgery, and bladder catheter was needed during the post operative period. Consequently, these comprehensive treatments were considered to be effective to prevent the repeated hyperammonemic encephalopathy.

## Conclusions

We herein experienced a rare case of hyperammonemic encephalopathy due to rectal cancer. Urinary diversion caused by rectovesical and ileal fistulae and urinary infection were the causative factors of hyperammonemic encephalopathy. This potential complication should therefore be considered in patients with vesicointestinal fistulae.

## Consent

Written informed consent was obtained from the family of patient for publication of this case and any accompanying images. A copy of the written consent is available for review by the Editor-in-Chief of this journal.
